# Supplementary material for: Inequalities and determinants of unmet need for SARS-CoV-2 testing in Ghana, Burkina Faso and Madagascar (2020 – 2021)
Source: Commun Med (Lond). 2026 May 15;6:282. doi: 10.1038/s43856-026-01637-z (PMC13179364; doi:10.1038/s43856-026-01637-z)
Supplement: Supplementary file 2 — Supplementary Information [file 43856_2026_1637_MOESM2_ESM.pdf]

Supplementary Information

Inequalities and determinants of unmet need for SARS-CoV-2 testing in Ghana, Burkina Faso and Madagascar (2020 – 2021)

Supplementary Figure 1A: Concentration curves for COVID-19 testing and unmet need in Burkina Faso Concentration curves showing the distribution of COVID-19 testing and unmet need across socioeconomic groups in Burkina Faso. Four panels display: testing uptake, serological-based unmet need (measure 1), exposure-based unmet need (measure 2), and symptom-based unmet need (measure 3). The diagonal line represents equality. Curves below indicate pro-rich distribution; curves above indicate pro-poor distribution. Testing is concentrated among wealthier populations; unmet need shows modest pro-poor inequality.

Supplementary Figure 1B: Concentration curves for COVID-19 testing and unmet need in Ghana Concentration curves showing the distribution of COVID-19 testing and unmet need across socioeconomic groups in Ghana. Four panels display: testing uptake, serological-based unmet need (measure 1), exposure-based unmet need (measure 2), and symptom-based unmet need (measure 3). The diagonal line represents equality. Curves below indicate pro-rich distribution; curves above indicate pro-poor distribution. Testing is concentrated among wealthier populations; unmet need shows modest pro-poor inequality.

Supplementary Figure 1C: Concentration curves for COVID-19 testing and unmet need in Madagascar Concentration curves showing the distribution of COVID-19 testing and unmet need across socioeconomic groups in Madagascar. Four panels display: testing uptake, serological-based unmet need (measure 1), exposure-based unmet need (measure 2), and symptom-based unmet need (measure 3). The diagonal line represents equality. Curves below indicate pro-rich distribution; curves above indicate pro-poor distribution. Testing is concentrated among wealthier populations; unmet need shows modest pro-poor inequality.

Burkina Faso

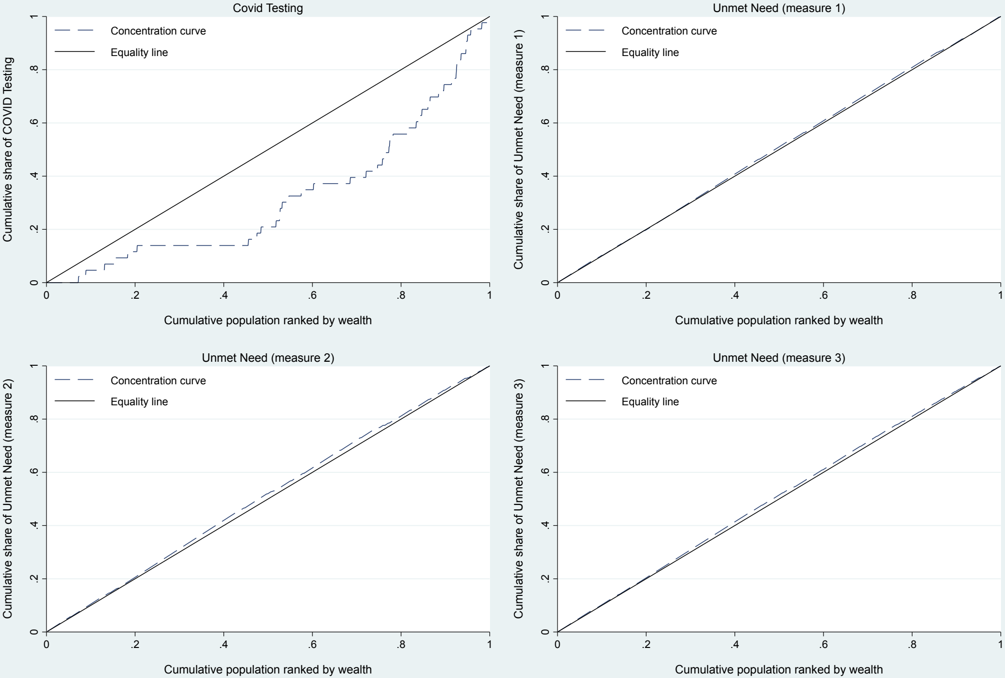

Ghana

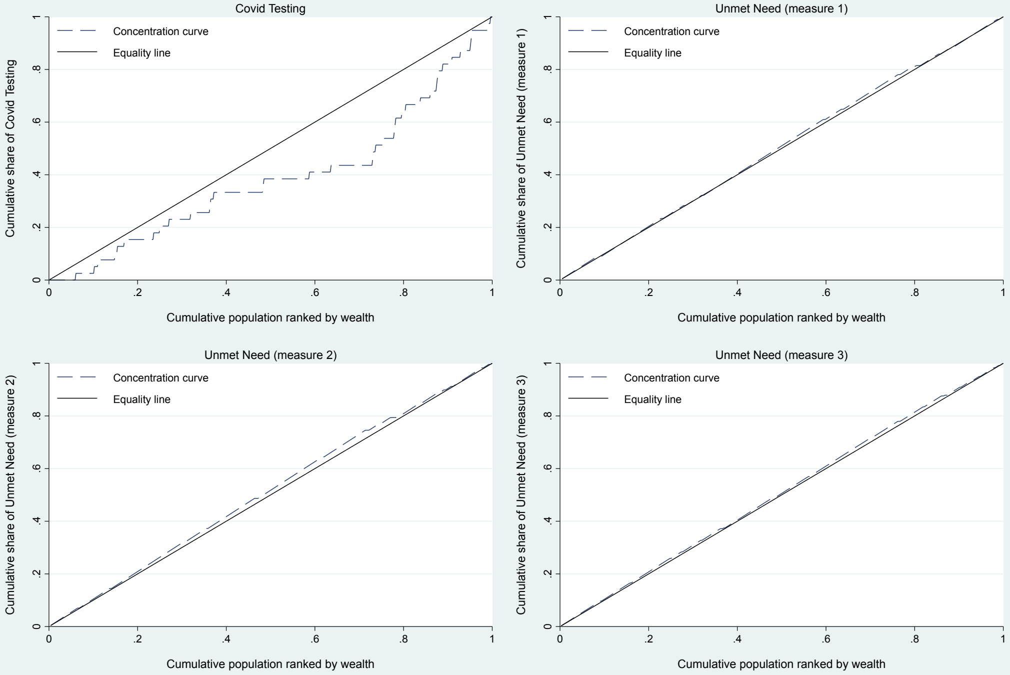

Covid Testing
Unmet Need (measure 1)
Unmet Need (measure 2)
Unmet Need (measure 3)
Concentration curve
Equality line
Cumulative share of Covid Testing
Cumulative share of Unmet Need (measure 1)
Cumulative share of Unmet Need (measure 2)
Cumulative share of Unmet Need (measure 3)
Cumulative population ranked by wealth
0
.2
.4
.6
.8
1

Madagascar

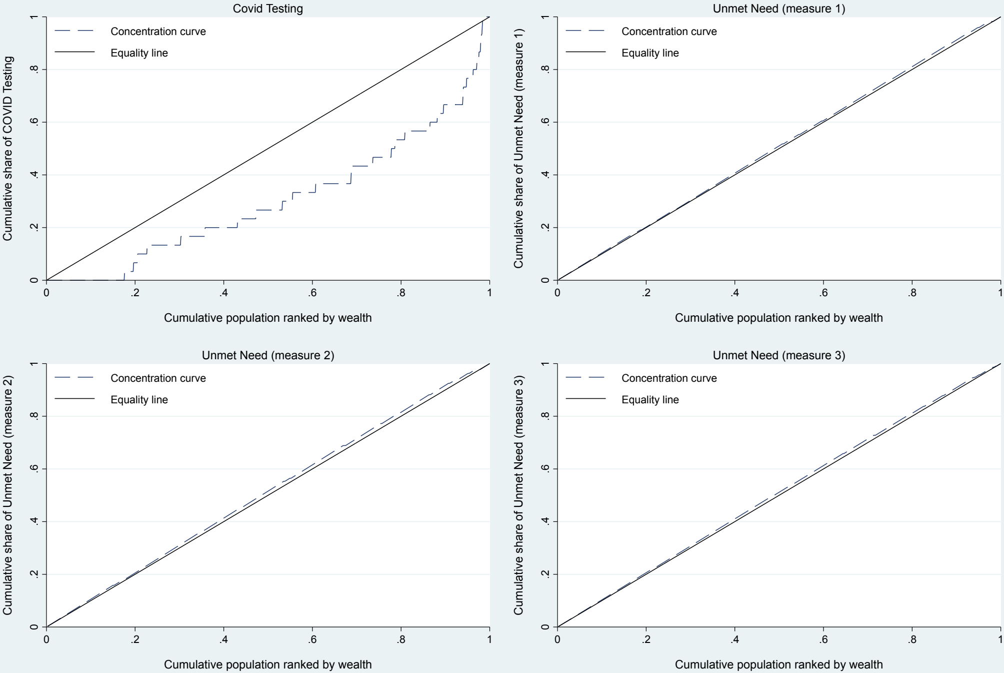

Covid Testing
Concentration curve
Equality line
Cumulative share of COVID Testing
0
.2
.4
.6
.8
1
Cumulative population ranked by wealth
Unmet Need (measure 1)
Cumulative share of Unmet Need (measure 1)
Unmet Need (measure 2)
Cumulative share of Unmet Need (measure 2)
Unmet Need (measure 3)
Cumulative share of Unmet Need (measure 3)
