# Supplementary material for: Inequalities and determinants of unmet need for SARS-CoV-2 testing in Ghana, Burkina Faso and Madagascar (2020 – 2021)
Source: Commun Med (Lond). 2026 May 15;6:282. doi: 10.1038/s43856-026-01637-z (PMC13179364; doi:10.1038/s43856-026-01637-z)
Supplement: Supplementary file 3 — Description of Additional Supplementary files [file 43856_2026_1637_MOESM3_ESM.docx]

Description of Additional Supplementary Files

Supplementary Data 1- Stata code for statistical analyses.

Supplementary Data 2- Source dataset saved as .csv-file.
