# Supplementary material for: Inequalities and determinants of unmet need for SARS-CoV-2 testing in Ghana, Burkina Faso and Madagascar (2020 – 2021)
Source: Commun Med (Lond). 2026 May 15;6:282. doi: 10.1038/s43856-026-01637-z (PMC13179364; doi:10.1038/s43856-026-01637-z)
Supplement: Supplementary file 4 — Supplementary Data 1 [file 43856_2026_1637_MOESM4_ESM.docx]

clear

set more off

*** Loading in data

global data "C:\Users\\`=c(username)'\OneDrive\SeroCov\Analysis\data"

*global results "C:\Users\\`=c(username)'\OneDrive\SeroCov\Analysis\results"

global results "/Users/jacobnovignon/Library/CloudStorage/OneDrive-Personal/SeroCov/Analysis/results"

*use "$data\all_countries", clear

use "/Users/jacobnovignon/Library/CloudStorage/OneDrive-Personal/SeroCov/2025/Review 2/all_countries_with_psu.dta", clear

/*****Selecting one random household member*****

set seed 12345

gen double rand = runiform()

bys screening_id (rand): keep if _n == 1

drop rand

*/

****** Univariate Analysis *******

foreach var of varlist wealth_quint1 age_cat daily {

tab `var', gen(`var'_)

}

*employment

** create variables to compare other wealth quintiles to the poorest

forvalues i=2/5 {

gen wq_`i'=.

replace wq_`i' = 1 if wealth_quint1 == `i'

replace wq_`i' = 0 if wealth_quint1 == 1

}

** create variables to compare daily contacts

forvalues i=2/4 {

gen dlycont_`i'=.

replace dlycont_`i' = 1 if daily_contacts == `i'

replace dlycont_`i' = 0 if daily_contacts == 1

}

** create variables to compare age categories

forvalues i=2/3 {

gen cage_`i'=.

replace cage_`i' = 1 if age_cat == `i'

replace cage_`i' = 0 if age_cat == 1

}

ttest underlying_cond, by(covid_test)

ttest risk, by(covid_test)

ttest travel, by(covid_test)

ttest wealth_quint1_1, by(covid_test)

ttest wealth_quint1_2, by(covid_test)

ttest wealth_quint1_3, by(covid_test)

ttest wealth_quint1_4, by(covid_test)

ttest wealth_quint1_5, by(covid_test)

ttest daily_contacts_1, by(covid_test)

ttest daily_contacts_2, by(covid_test)

ttest daily_contacts_3, by(covid_test)

ttest daily_contacts_4, by(covid_test)

ttest age_cat_1 , by(covid_test)

ttest age_cat_2, by(covid_test)

ttest age_cat_3, by(covid_test)

ttest sex, by(covid_test)

ttest employ_status, by(covid_test)

ttest underlying_cond, by(unmet_need1)

ttest risk, by(unmet_need1)

ttest travel, by(unmet_need1)

ttest wealth_quint1_1, by(unmet_need1)

ttest wealth_quint1_2, by(unmet_need1)

ttest wealth_quint1_3, by(unmet_need1)

ttest wealth_quint1_4, by(unmet_need1)

ttest wealth_quint1_5, by(unmet_need1)

ttest sex, by(unmet_need1)

ttest daily_contacts_1, by(unmet_need1)

ttest daily_contacts_2, by(unmet_need1)

ttest daily_contacts_3, by(unmet_need1)

ttest daily_contacts_4, by(unmet_need1)

ttest age_cat_1 , by(unmet_need1)

ttest age_cat_2 , by(unmet_need1)

ttest age_cat_3 , by(unmet_need1)

ttest employ_status, by(unmet_need1)

ttest underlying_cond, by(unmet_need2a)

ttest risk, by(unmet_need2a)

ttest travel, by(unmet_need2a)

ttest wealth_quint1_1, by(unmet_need2a)

ttest wealth_quint1_2, by(unmet_need2a)

ttest wealth_quint1_3, by(unmet_need2a)

ttest wealth_quint1_4, by(unmet_need2a)

ttest wealth_quint1_5, by(unmet_need2a)

ttest sex, by(unmet_need2a)

ttest daily_contacts_1, by(unmet_need2a)

ttest daily_contacts_2, by(unmet_need2a)

ttest daily_contacts_3, by(unmet_need2a)

ttest daily_contacts_4, by(unmet_need2a)

ttest age_cat_1 , by(unmet_need2a)

ttest age_cat_2 , by(unmet_need2a)

ttest age_cat_3 , by(unmet_need2a)

ttest employ_status, by(unmet_need2a)

ttest underlying_cond, by(unmet_need2b)

ttest risk, by(unmet_need2b)

ttest travel, by(unmet_need2b)

ttest wealth_quint1_1, by(unmet_need2b)

ttest wealth_quint1_2, by(unmet_need2b)

ttest wealth_quint1_3, by(unmet_need2b)

ttest wealth_quint1_4, by(unmet_need2b)

ttest wealth_quint1_5, by(unmet_need2b)

ttest sex, by(unmet_need2b)

ttest daily_contacts_1, by(unmet_need2b)

ttest daily_contacts_2, by(unmet_need2b)

ttest daily_contacts_3, by(unmet_need2b)

ttest daily_contacts_4, by(unmet_need2b)

ttest age_cat_1 , by(unmet_need2b)

ttest age_cat_2 , by(unmet_need2b)

ttest age_cat_3 , by(unmet_need2b)

ttest employ_status, by(unmet_need2b)

****** Bivariate Analysis *******

ttest covid_test, by(underlying_cond)

ttest covid_test, by(travel)

ttest covid_test, by(risk)

ttest covid_test, by(wq_2)

ttest covid_test, by(wq_3)

ttest covid_test, by(wq_4)

ttest covid_test, by(wq_5)

ttest covid_test, by(sex)

ttest covid_test, by(dlycont_2)

ttest covid_test, by(dlycont_3)

ttest covid_test, by(dlycont_4)

ttest covid_test, by(cage_2)

ttest covid_test, by(cage_3)

ttest covid_test, by(employ_status)

ttest unmet_need1, by(underlying_cond)

ttest unmet_need1, by(travel)

ttest unmet_need1, by(risk)

ttest unmet_need1, by(wq_2)

ttest unmet_need1, by(wq_3)

ttest unmet_need1, by(wq_4)

ttest unmet_need1, by(wq_5)

ttest unmet_need1, by(sex)

ttest unmet_need1, by(dlycont_2)

ttest unmet_need1, by(dlycont_3)

ttest unmet_need1, by(dlycont_4)

ttest unmet_need1, by(cage_2)

ttest unmet_need1, by(cage_3)

ttest unmet_need1, by(employ_status)

ttest unmet_need2a, by(underlying_cond)

ttest unmet_need2a, by(travel)

ttest unmet_need2a, by(risk)

ttest unmet_need2a, by(wq_2)

ttest unmet_need2a, by(wq_3)

ttest unmet_need2a, by(wq_4)

ttest unmet_need2a, by(wq_5)

ttest unmet_need2a, by(sex)

ttest unmet_need2a, by(dlycont_2)

ttest unmet_need2a, by(dlycont_3)

ttest unmet_need2a, by(dlycont_4)

ttest unmet_need2a, by(cage_2)

ttest unmet_need2a, by(cage_3)

ttest unmet_need2a, by(employ_status)

ttest unmet_need2b, by(underlying_cond)

ttest unmet_need2b, by(travel)

ttest unmet_need2b, by(risk)

ttest unmet_need2b, by(wq_2)

ttest unmet_need2b, by(wq_3)

ttest unmet_need2b, by(wq_4)

ttest unmet_need2b, by(wq_5)

ttest unmet_need2b, by(sex)

ttest unmet_need2b, by(dlycont_2)

ttest unmet_need2b, by(dlycont_3)

ttest unmet_need2b, by(dlycont_4)

ttest unmet_need2b, by(cage_2)

ttest unmet_need2b, by(cage_3)

ttest unmet_need2b, by(employ_status)

lab def contacts 1 "Less than 5" 2 "5 to less than 10" 3 "10 to less than 50" 4 "50 or more", replace

lab val daily_contacts contacts

global y covid_test unmet_need1 unmet_need2a unmet_need2b antibody

global x1 wealth_quint1_1-wealth_quint1_5 sex underlying_cond daily_contacts_1-daily_contacts_4 travel hhsize risk_perception age_cat_1-age_cat_3 eduyrs employ_status

global asset_vars h11_rooms_s own_hsestatus1 own_hsestatus2 own_hsestatus3 own_hsestatus4 own_hsestatus5 toilet_facility1 toilet_facility2 toilet_facility3 toilet_facility4 toilet_facility5 cooking1 cooking2 cooking3 cooking4 lighting1 lighting2 lighting3 lighting4 rainy_water1 rainy_water2 rainy_water3 rainy_water4 dry_water1 dry_water2 dry_water3 dry_water4 dry_water5 floor1 floor2 floor3 floor4 floor5 floor6 floor7 floor8 roof1 roof2 roof3 roof4 roof5 walls1 walls2 walls3 h21_amenradio_d h22_amentv_d h23_amenplayer_d h24_amenlandline_d h25_amenmobile_d h26_amenfridgefreezer_d h27_amenestove_d h28_amengasstove_d h34_car_d h35_amendmotorbike_d h37_amendbicycle_d h38_computer_d h39_laptop_d

asdoc sum $y $x1

tabstat daily_contacts hhsize eduyrs, stat(median iqr)

asdoc sum $asset_vars

*logistic covid_test SES_Index1 sex underly travel risk i.daily_contacts hhsize m01_2_ageyears_q eduyrs employ_status i.country, vce(clust screening_id)

logistic covid_test i.wealth_quint1 sex underly travel risk i.daily_contacts hhsize m01_2_ageyears_q eduyrs employ_status i.country, vce(clust psu_region)

est sto covidtest

logistic unmet_need1 i.wealth_quint1 sex underly travel risk i.daily_contacts hhsize m01_2_ageyears_q eduyrs employ_status i.country, vce(clust psu_region)

est sto need1

logistic unmet_need2a i.wealth_quint1 sex underly travel risk i.daily_contacts hhsize m01_2_ageyears_q eduyrs employ_status i.country, vce(clust psu_region)

est sto need2a

logistic unmet_need2b i.wealth_quint1 sex underly travel risk i.daily_contacts hhsize m01_2_ageyears_q eduyrs employ_status i.country, vce(clust psu_region)

est sto need2b

#delimit;

esttab covidtest need1 need2a need2b

using "$results/all_unmetneed_CI_26.rtf", replace

eform cells(b(star fmt(3)) ci(par fmt(3))) constant style(fixed)

starlevels("*" 0.10 "**" 0.05 "***" 0.01) nonum stats(N)

title(Unmet Need for Covid Testing in All Countries)

scalar(r2_p "Pseudo R-squared")

nogap compress legend

;

logistic covid_test i.wealth_quint1 sex underly travel risk i.daily_contacts hhsize m01_2_ageyears_q employ_status i.country, vce(clust psu_region)

est sto covidtest

logistic unmet_need1 i.wealth_quint1 sex underly travel risk i.daily_contacts hhsize m01_2_ageyears_q employ_status i.country, vce(clust psu_region)

est sto need1

logistic unmet_need2a i.wealth_quint1 sex underly travel risk i.daily_contacts hhsize m01_2_ageyears_q employ_status i.country, vce(clust psu_region)

est sto need2a

logistic unmet_need2b i.wealth_quint1 sex underly travel risk i.daily_contacts hhsize m01_2_ageyears_q employ_status i.country, vce(clust psu_region)

est sto need2b

#delimit;

esttab covidtest need1 need2a need2b

using "$results/all_unmetneed_CI_26_noeduc.rtf", replace

eform cells(b(star fmt(3)) ci(par fmt(3))) constant style(fixed)

starlevels("*" 0.10 "**" 0.05 "***" 0.01) nonum stats(N)

title(Unmet Need for Covid Testing in All Countries)

scalar(r2_p "Pseudo R-squared")

nogap compress legend

;

**** Socioeconomic related inequality analysis

foreach var of global y {

conindex `var', rank(SES_Index1) limits(0 1) erreygers bounded clust(psu_region)

est sto `var'

local ci_estimate = r(CI) // Store the point estimate

local se = r(CIse) // Store the standard error

local lb = `ci_estimate' - 1.96 * `se' // Lower bound

local ub = `ci_estimate' + 1.96 * `se' // Upper bound

di "95% CI: [`lb', `ub']"

}

coefplot covid_test || unmet_need1 || unmet_need2a || unmet_need2b, ///

keep(__00001W) ///

vertical ///

xtitle("") ///

ytitle("Concentration index with 95% CI") ///

bycoefs byopts(yrescale) ///

ciopts(recast(rcap)) ///

recast(scatter) ///

citop ///

yline(0, lcolor(gs8)) ///

xlabel( ///

1 "COVID testing (n=2741)" ///

2 "Unmet need measure 1 (n=1086)" ///

3 "Unmet need measure 2 (n=1117)" ///

4 "Unmet need measure 3 (n=1693)", ///

labsize(vsmall)) ///

ylabel(-0.300(0.100)0.300, gmax angle(horizontal)) ///

mlabel format(%9.3f) mlabcolor(black) mlabgap(*1) mlabposition(3) ///

levels(95 95) ///

mcolor(navy8) msize(medium) msymbol(circle) ///

title("Concentration indices for outcomes", size(medium) color(black)) ///

graphregion(color(white) margin(b+12)) ///

plotregion(color(white)) ///

xsize(10) ysize(5)

graph save "$results\Coefplots", replace

graph export "$results\Coefplots.tif", replace

foreach var of varlist covid_test unmet_need1 unmet_need2a unmet_need2b {

glcurve `var', glvar(yord) pvar(rank) sortvar(SES_Index1) replace lorenz nograph

gen rank2=rank

lab var rank2 "line of equality"

lab var rank "cumulative share of individuals(poorest to richest)"

twoway (line yord rank, sort clwidth(medthin) clcolor(navy8)) ///

(line rank2 rank , sort clwidth(medthin) clcolor(black)) ///

, ytitle(Cumulative share of `var', size(medium)) ///

yscale(titlegap(5)) xtitle(Cumulative share of individuals (poorest to richest), size(medium)) legend(rows(5)) xscale(titlegap(5)) ///

legend(position(11) ring(0) region(lwidth(none))) plotregion(margin(zero)) ysize(5.75) xsize(5) plotregion(lcolor(none)) ///

title(Concentration curve: `var', size (small) justification(center) color (black)) saving(all_`var', replace)

drop rank2 rank yord

}

graph combine all_covid_test.gph all_unmet_need1.gph all_unmet_need2a.gph all_unmet_need2b.gph, rows(3) iscale(.4)

graph export "$results\all_combined.tif", replace

foreach var of varlist covid_test unmet_need1 unmet_need2a unmet_need2b {

glcurve `var' if country==1, glvar(yord) pvar(rank) sortvar(SES_Index1) replace lorenz nograph

gen rank2=rank

lab var rank2 "line of equality"

lab var rank "cumulative share of individuals(poorest to richest)"

twoway (line yord rank, sort clwidth(medthin) clcolor(navy8)) ///

(line rank2 rank , sort clwidth(medthin) clcolor(black)) ///

, ytitle(Cumulative share of `var', size(medium)) ///

yscale(titlegap(5)) xtitle(Cumulative share of individuals (poorest to richest), size(medium)) legend(rows(5)) xscale(titlegap(5)) ///

legend(position(11) ring(0) region(lwidth(none))) plotregion(margin(zero)) ysize(5.75) xsize(5) plotregion(lcolor(none)) ///

title(Concentration curve: `var', size (small) justification(center) color (black)) saving(all_`var', replace)

drop rank2 rank yord

}

graph combine all_covid_test.gph all_unmet_need1.gph all_unmet_need2a.gph all_unmet_need2b.gph, rows(3) iscale(.4)

graph export "$results\all_combined_bf.tif", replace

foreach var of varlist covid_test unmet_need1 unmet_need2a unmet_need2b {

glcurve `var' if country==2, glvar(yord) pvar(rank) sortvar(SES_Index1) replace lorenz nograph

gen rank2=rank

lab var rank2 "line of equality"

lab var rank "cumulative share of individuals(poorest to richest)"

twoway (line yord rank, sort clwidth(medthin) clcolor(navy8)) ///

(line rank2 rank , sort clwidth(medthin) clcolor(black)) ///

, ytitle(Cumulative share of `var', size(medium)) ///

yscale(titlegap(5)) xtitle(Cumulative share of individuals (poorest to richest), size(medium)) legend(rows(5)) xscale(titlegap(5)) ///

legend(position(11) ring(0) region(lwidth(none))) plotregion(margin(zero)) ysize(5.75) xsize(5) plotregion(lcolor(none)) ///

title(Concentration curve: `var', size (small) justification(center) color (black)) saving(all_`var', replace)

drop rank2 rank yord

}

graph combine all_covid_test.gph all_unmet_need1.gph all_unmet_need2a.gph all_unmet_need2b.gph, rows(3) iscale(.4)

graph export "$results\all_combined_gh.tif", replace

foreach var of varlist covid_test unmet_need1 unmet_need2a unmet_need2b {

glcurve `var' if country==3, glvar(yord) pvar(rank) sortvar(SES_Index1) replace lorenz nograph

gen rank2=rank

lab var rank2 "line of equality"

lab var rank "cumulative share of individuals(poorest to richest)"

twoway (line yord rank, sort clwidth(medthin) clcolor(navy8)) ///

(line rank2 rank , sort clwidth(medthin) clcolor(black)) ///

, ytitle(Cumulative share of `var', size(medium)) ///

yscale(titlegap(5)) xtitle(Cumulative share of individuals (poorest to richest), size(medium)) legend(rows(5)) xscale(titlegap(5)) ///

legend(position(11) ring(0) region(lwidth(none))) plotregion(margin(zero)) ysize(5.75) xsize(5) plotregion(lcolor(none)) ///

title(Concentration curve: `var', size (small) justification(center) color (black)) saving(all_`var', replace)

drop rank2 rank yord

}

graph combine all_covid_test.gph all_unmet_need1.gph all_unmet_need2a.gph all_unmet_need2b.gph, rows(3) iscale(.4)

graph export "$results\all_combined_mg.tif", replace

********** 2025 **********

logistic covid_test i.wealth_quint1 sex underly travel risk i.daily_contacts hhsize m01_2_ageyears_q employ_status i.country, vce(clust screening_id)

est sto covidtest

logistic unmet_need1 i.wealth_quint1 sex underly travel risk i.daily_contacts hhsize m01_2_ageyears_q employ_status i.country, vce(clust screening_id)

est sto need1

logistic unmet_need2a i.wealth_quint1 sex underly travel risk i.daily_contacts hhsize m01_2_ageyears_q employ_status i.country, vce(clust screening_id)

est sto need2a

logistic unmet_need2b i.wealth_quint1 sex underly travel risk i.daily_contacts hhsize m01_2_ageyears_q employ_status i.country, vce(clust screening_id)

est sto need2b

#delimit;

esttab covidtest need1 need2a need2b

using "$results\all_unmetneed_Quintiles_25.rtf", replace

eform cells(b(star fmt(3)) ci(par fmt(3))) constant style(fixed)

starlevels("*" 0.10 "**" 0.05 "***" 0.01) nonum stats(N)

title(Unmet Need for Covid Testing in All Countries)

scalar(r2_p "Pseudo R-squared")

nogap compress legend

;
